# Metabolic adaptation of wheat grain contributes to a stable filling rate under heat stress

**DOI:** 10.1093/jxb/ery303

**Published:** 2018-08-24

**Authors:** Xiaoming Wang, Lijiang Hou, Yunze Lu, Bingjin Wu, Xue Gong, Manshuang Liu, Jun Wang, Qixin Sun, Elizabeth Vierling, Shengbao Xu

**Affiliations:** 1 State Key Laboratory of Crop Stress Biology for Arid Areas, College of Agronomy, Northwest A&F University, Yangling, Shaanxi, China; 2 Department of Plant Genetics & Breeding, China Agricultural University, Haidian District, Beijing, China; 3 Department of Biochemistry & Molecular Biology, University of Massachusetts, Amherst, MA, USA

**Keywords:** Grain filling, heat stress, heat adaptive metabolism, metabolomics, proteomics, reserves deposition, wheat (*Triticum aestivum*)

## Abstract

Wheat (*Triticum aestivum*) is particularly vulnerable to heat stress during the grain filling stage, and this can adversely affect the final yield. However, the underlying physiological and molecular mechanisms are largely unknown. In this study, the effects of heat stress on grain filling were investigated using wheat varieties with different levels of thermotolerance. Decreased grain weights and filling durations, increased protein contents, and stable filling rates across diverse varieties under different heat regimes suggested a general mechanism for heat adaptation. Proteomic analysis identified 309 heat-responsive proteins (HRPs), and revealed a general decrease in protein synthesis components and metabolic proteins, but a significant increase in stress-response proteins and storage proteins. Metabolomic analysis identified 98 metabolites specifically changed by heat stress, and suggested a global decrease in the content of carbohydrate metabolites, an increased content of amino acids, and stable levels of starch synthesis precursors. The energy-consuming HRPs suggested that less energy was channelled into metabolism and protein synthesis, whereas more energy was allocated to the stress response under elevated heat conditions. Collectively, the data demonstrated a widely distributed mechanism for heat adaptation of metabolism, in which the assimilation and energy required for metabolism and protein synthesis are reallocated to heat protection and deposition of reserves, resulting in increased storage protein accumulation and a stable filling rate.

## Teaser

When wheat is subjected to heat stress during grain filling, metabolic adaptation reallocates resources to deposition of reserves and prioritizes a stable filling rate.

## Introduction

Wheat (*Triticum aestivum* L.), a major staple food resource for the world’s population, is vulnerable to heat stress (Semenov and Shewry, 2011; Lobell and Tebaldi, 2014; Tack *et al.*, 2015; Tao *et al.*, 2015; Scafaro and Atkin, 2016). In most wheat-growing areas, the crop typically experiences the highest temperatures during the grain-filling stage of its life cycle. Heat stress during this stage usually causes a severe reduction in grain weight and a corresponding decrease in yield (Wollenweber *et al.*, 2003; ur Rehman *et al.*, 2009; Farooq *et al.*, 2011; Tack *et al.*, 2015; Lesk *et al.*, 2016; Scafaro and Atkin, 2016).

Increasing evidence suggests that a decrease in the filling duration is the major reason for the reduction in wheat grain weight caused by heat stress (Zhao *et al.*, 2007; Chauhan *et al.*, 2009; Farooq *et al.*, 2011). However, detailed studies have demonstrated that high temperature impedes the synthesis of starch, the major reserve in wheat grains (constituting ~75% of grain weight), by reducing the expression and activity of starch synthesis-related proteins (Blumenthal *et al.*, 1994; Keeling *et al.*, 1994; Skylas *et al.*, 2002; Zahedi *et al.*, 2003; Majoul *et al.*, 2004; Lin *et al.*, 2005; Yamakawa and Hakata, 2010; Mangelsen *et al.*, 2011; Liao *et al.*, 2014). Hence, reduced starch synthesis has also been proposed to be the main reason for heat stress-induced reduction in grain weight. Arguments about the relative importance of the filling duration versus starch synthesis may reflect the complexity of the damage caused by heat, with distinct effects being observed for different heat-resistant varieties and different heat regimes (Farooq *et al.*, 2011). Thus, further investigations with more wheat varieties under different heat regimes are needed to determine whether there are any general heat effects on the grain filling process.

Despite the significant decrease in starch synthesis-related proteins, grains can maintain a stable or even higher filling rate during heat stress (Zhao *et al.*, 2007; Farooq *et al.*, 2011). It is assumed that the accumulation of more storage proteins, another major reserve in grains (~20%), during heat stress (Wardlaw and Wrigley, 1994; Wrigley *et al.*, 1994; Wardlaw *et al.*, 2002) is a critical mechanism to compensate for the reduction in starch deposition and hence to maintain a stable filling rate, as assessed by weight changes (Farooq *et al.*, 2011). However, the compensatory mechanism that leads to the altered metabolism in filling grains is largely unknown.

Transcriptome analyses have determined that thousands of genes are altered in response to heat stress in rice caryopses (Yamakawa *et al.*, 2007; Yamakawa and Hakata, 2010) and barley grains (Mangelsen *et al.*, 2011). These studies point to global metabolic changes in response to heat stress, including a general increase in heat-shock proteins (HSPs), depression of starch synthesis, and a decrease in protein synthesis. Proteomic analyses have consistently shown that HSPs are dramatically induced during heat stress in filling wheat grains (Skylas *et al.*, 2002; Majoul *et al.*, 2004; Neilson *et al.*, 2010) and rice caryopses (Liao *et al.*, 2014), while the key proteins for starch synthesis, including glucose-1-phosphate adenyltransferase (AGPase) and granule-bound starch synthetase, show significant decreases (Zahedi *et al.*, 2003; Majoul *et al.*, 2004; Neilson *et al.*, 2010), supporting a decrease in starch synthesis during heat stress. Furthermore, proteomic evidence has also indicated that there is impaired ATP and protein synthesis in heat-stressed wheat grains (Majoul *et al.*, 2004; Li *et al.*, 2011). These proteomic results have contributed valuable data towards understand the effects of heat stress and the underlying molecular basis for changes in grain filling. However, only a limited number of HRPs were identified because of the limitations of the 2-D gel-based proteomics approach used in these previous studies. A more powerful method and comprehensive analysis of HRPs is thus required to clarify the alterations that occur in filling grains subject to heat stress.

Detailed investigation of metabolites has also provided important clues towards determining metabolic responses to heat, especially when combined with transcriptomic and proteomic analyses. Several specific metabolic alterations have been documented, including decreased tricarboxylic acid (TCA) cycle intermediates and dramatically increased amino acid content in heat-stressed rice caryopses (Yamakawa and Hakata, 2010) and barley grains (Mangelsen *et al.*, 2011). These investigations have demonstrated changes in corresponding pathways, but have usually miss the connections that co-ordinate responses among different pathways during heat stress. Recent developments in metabolomics mean that it is now possible to gain comprehensive insights into metabolism and have provided the power to identify the phenotypic effects of abiotic stresses on plants (Brunetti *et al.*, 2013). Such an approach has been applied to analyse changes in lipid metabolites in heat-stressed wheat leaves (Narayanan *et al.*, 2016*a*, 2016*b*). When combined with proteomic analysis, metabolite data can provide evidence for co-ordinated metabolic changes (Kaspar-Schoenefeld *et al.*, 2016), thus potentially clarifying general mechanisms by which metabolism adapts to heat during grain filling.

A filling grain is a typical low-oxygen organ that restricts its energy metabolism (Geigenberger, 2003). This limited energy supply determines the status of starch and protein synthesis in the grain (Xu *et al.*, 2008, 2010; Lee *et al.*, 2015), and it has been regarded as a basic factor for evaluating metabolic alterations. Importantly, allocating this limited energy plays a critical role in determining how assimilation is channelled into different pathways and the resulting composition of different reserves (Rolletschek *et al.*, 2004). Detailed studies have determined that the TCA cycle and ATP synthesis are significantly decreased in filling grain in response to heat stress (Majoul *et al.*, 2004; Yamakawa and Hakata, 2010; Li *et al.*, 2011; Mangelsen *et al.*, 2011). However, little is known about the channeling of energy under heat stress, and this hinders our understanding of metabolic alterations and assimilation partitioning in filling grains subjected to stress.

In this study, a diverse group of wheat accessions were subjected to heat stress in the field during the grain-filling stage in order to determine the general effects on grain weight, protein content, and filling rate. Growth chamber experiments were then performed to further examine proteomic and metabolomic changes induced by heat stress during grain filling. Using this approach, the underlying physiological and molecular mechanisms induced by heat stress in filling wheat grains were investigated.

## Materials and methods

### Field investigation

We conducted a preliminary evaluation of thermotolerance on 237 spring wheat accessions obtained from the International Center for Agricultural Research in the Dry Areas (ICARDA, Rabat, Morocco) and selected 36 for further study. Two additional spring wheat varieties, Liao-10 and Chinese Spring, were also studied (see Supplementary Table S1 at *JXB* online). The accessions (all hexaploid wheat, AABBDD) were planted at Northwest A&F University in Yangling, China (34°28′N, 108°07′E, altitude 517 m) with different sowing times in two growing seasons. In the 2015–16 season, the early and late sowings took place on October 8th 2015 and February 18th 2016, respectively. In the 2016–17 season, the early and late sowings took place on October 18th 2016 and February 9th 2017, respectively. For each variety/accession, 40 seeds were planted per row (2 m in length, 0.25 m apart), and nine replicate rows were planted at each sowing time. The flowering date was recorded as the time when 50% of the main spikes were flowering, and the maturity date was recorded as the time when 95% of the spikes had lost their green colour. The filling duration was calculated as the time between flowering and maturity. Twenty comparable main spikes were collected from each row for determination of grain weight using an automatic grain test instrument (Wanshen SC-G, China) and determination of protein content using a Model DA 7250 near-infrared analyser (Perten Instruments, Hägersten, Sweden). The filling rate was calculated as: (grain weight)/(filling duration).

### Plant material, sampling, and growth conditions for proteomic and metabolomic analyses

Plants of the variety Chinese Spring were cultured in 25-cm pots at a density of 16 plants per pot in a greenhouse at a temperature of 15–30 °C. Plants were watered once a week, and artificial light was applied when there were fewer than 12 h of natural daylight. The flowering date of each spike was recorded as the time when the first flower appeared on the spike. Pots with comparable plants (i.e. similar flowering time and growth status) were selected at 12 d after flowering (DAF) and transferred into growth chambers with a 24/17 °C, 14/10 h day/night cycle. The photosynthetic photon flux density was 360 µmol m^–2^ s^–1^ at canopy height, and relative humidity ranged from 40% to 70%. After 3 d of adaptation to the growth-chamber environment, half of the plants (~50) were transferred to heat-stress conditions (37/17 °C day/night, other conditions the same), while the others were maintained under the standard conditions as a control. After three day/night cycles, the heat-stressed plants were sampled at 4 h into the light period, and control plants were sampled at the same time for metabolomic and proteomic analyses. A minimum of 20 spikes were collected for each treatment. The remaining plants were kept in the growth chambers until mature grains could be sampled. Samples for analysis of free amino acid (AA) content were collected on the first day of heat stress (15 DAF). The experiment was repeated three times and similar results were obtained. Mature grains from control and treated plants were analysed for storage protein components by SDS–PAGE and MS analysis.

To investigate heat responses at the transcriptional level, seven accessions with different levels of thermotolerance (Supplementary Table S1) were selected at the filling stage (around 18 DAF) from the field in the morning when the air temperature was about 22 °C. Plants were transferred into 25-cm pots filled with soil, and then placed in the growth chambers at either 24 °C or 37 °C for 4 h. Samples of grains from three independent plant were then taken for mRNA extraction.

### Isobaric tandem mass tag (TMT)-labelled quantitative proteomic analysis

Protein extraction was performed on samples of 12–15 filling grains (~500 mg) from a single spike. Three replicate spikes from each treatment were used for proteomic analysis. Samples were ground in liquid nitrogen and collected in 5-ml centrifuge tubes. They were then sonicated three times on ice in lysis buffer [8 M urea, 2 mM EDTA, 10 mM DTT, and 1% protease inhibitor cocktail (Roche)] using a high-intensity ultrasonic processor (Scientz, Ningbo, China). The remaining debris was removed by centrifugation at 20000 *g* for 10 min at 4 °C. Protein was precipitated with cold 15% trichloroacetic acid-acetone for 2 h at –20 °C. After centrifugation at 20000 *g* for 10 min at 4 °C, the supernatant was discarded. The remaining precipitate was washed three times with cold acetone. The protein was redissolved in buffer [8 M urea and 100 mM triethylammonium bicarbonate (TEAB), pH 8.0]. The protein concentration was determined using a 2-D Quant kit following the manufacturer’s instructions (GE Healthcare).

#### Trypsin digestion and TMT labeling

The protein solutions were reduced with 10 mM DTT for 1 h at 37 °C and alkylated with 20 mM iodoacetamide for 45 min at room temperature in the dark. The protein samples (~100 μg) were then diluted by adding 100 mM TEAB to a urea concentration less than 2 M. Next, trypsin (Roche) was added at a 1:50 ratio (trypsin:protein mass) for a first digestion overnight, and at a 1:100 ratio for a second 4-h digestion, both at 37 °C. After digestion, the peptides were desalted using a Strata X C18 SPE column (Phenomenex, Torrance, CA, USA) and vacuum dried. The peptides were reconstituted in 0.5 M TEAB and processed using a TMTsixplex™ kit (ThermoFisher Scientific) according to the manufacturer’s instructions. The quantity of protein in each sample was presented basy the ratio, which is the abundance of a protein in a given sample compared to the average abundance of this protein in all six samples, and followed normalization as described by Rauniyar *et al.* (2013).

#### HPLC fractionation, LC-MS/MS analysis, and database searches

These procedures were performed as described by Lu *et al.* (2017). Tandem mass spectra were searched against local Triticum_aestivum.TGACv1.pep.all.fa downloaded from ftp://ftp.ensemblgenomes.org/pub/release-39/plants/fasta/triticum_aestivum/pep/Triticum_aestivum.TGACv1.pep.all.fa.gz. The MS data had been deposited to the ProteomeXchange Consortium via the PRIDE database (Vizcaíno *et al.*, 2014) partner repository with the dataset identifier PXD010340 and doi:10.6019/PXD010340.

### Separation of grain storage proteins and LC-MS/MS identification

Four mature grains were ground at room temperature. Total protein was extracted by vortexing for 15 min with 500 µl of extraction buffer [0.5 M Tris–HCl, pH 6.8, containing 0.4% (w/v) iodoacetamide and 1% (w/v) DTT], followed by centrifugation at 18000 *g* for 20 min. The supernatant was supplemented with four volumes of ice-cold 100% acetone to precipitate the proteins at –20 °C for 1 h, and they were collected by centrifugation at 18000 *g* for 20 min. After two washes with 80% ice-cold acetone, the resulting proteins were dried at room temperature prior to quantification and separation by SDS–PAGE. For both the control and heat stress treatments, four independent samples from two separate experiments were measured. In-gel protein digestion was performed as described by Xu *et al.* (2010), and then HPLC fractionation, LC-MS/MS analysis, and database searches were performed as described by Lu *et al.* (2017).

### Metabolomic analysis

Metabolite profiling was performed as described by Kind *et al.* (2009). Metabolite extraction was performed on 12–15 filling grains (~500 mg) from one spike, and six replicate spikes were analysed for the control and heat-stress treatments. A fraction enriched in primary metabolites was prepared for analysis by GC coupled with electron-impact ionization time-of-flight MS. In detail, 50 mg of ground grain material from one spike was collected and transferred into a 2-ml tube. Then, 60 µl of ribitol (0.2 mg ml^–1^ stock in dH_2_O) was added as an internal standard, followed by 0.5 ml of extraction liquid (methanol:chloroform, 3:1). To homogenize the tissue, one 5-mm and two 2-mm steel balls were added into the sample tubes, and samples were homogenized in a ball mill for 6 min at 55 Hz, followed by a 10-min incubation at 70 °C before centrifuging for 15 min at 15000 *g* at 4 °C. Then, 0.4 ml of supernatant was transferred into a fresh 2-ml GC–MS glass vial, dried in a vacuum concentrator without heating, and mixed with 80 μl of methoxyamination reagent (20 mg ml^–1^ in pyridine). After shaking for 2 h at 37 °C, 0.1 ml of BSTFA reagent [99:1 N,O-(bistrimethylsilyl) trifluoroacetamide: trimethylchlorosilane] was added, followed by further shaking for 1 h at 70 °C. The samples were then returned to room temperature and 10 µl of FAMEs (a standard mixture of fatty acid methyl esters: C8–C16, 1 mg ml^–1^; C18–C30, 0.5 mg ml^–1^ in chloroform; Dr. Ehrenstorfer GmbH) was added to each sample.

GC time-of-flight MS analysis was performed using a 7890 Gas Chromatograph System (Agilent Technologies) coupled with a Pegasus HT time-of-flight mass spectrometer (Leco Corp., St Joseph, MI, USA). The system utilized a DB-5MS capillary column coated with 5% diphenyl cross-linked with 95% dimethylpolysiloxane (30 m×250 μm inner diameter, 0.25 μm film thickness; J&W Scientific, Folsom, CA, USA). A 1-μl aliquot of the analyte was injected in splitless mode. Helium was used as the carrier gas, the front inlet purge flow was 3 ml min^–1^ and the gas flow rate through the column was 20 ml min^–1^. The initial temperature was kept at 50 °C for 1 min, raised to 330 °C at a rate of 10 °C min^–1^, and then kept at 330 °C for 5 min. The injection, transfer-line, and ion-source temperatures were 280, 280, and 220 °C, respectively. The energy was –70 eV in the electron-impact mode. The MS data were acquired in full-scan mode within the *m*/*z* range of 85–600 at a rate of 20 spectra s^–1^ after a solvent delay of 366 s. Six replicates for both heat stress and control conditions were analysed.

Chroma TOF 4.3X software (Leco Corp.) and the Leco-Fiehn Rtx5 database were used for raw peak extraction, data baseline filtering and baseline calibration, peak alignment, deconvolution analysis, peak identification, and integration of the peak area. The retention time index (RI) was used for peak identification. Usually, a peak generated by MS can hit multiple metabolite candidates, so the candidates that deviated more than 5000 against to RI reference were removed, then the candidate with highest similarity was selected as the identification result.

Only metabolites identified more than seven times in all replicated experiments (12 replicates, including six control and six heat treatments) were assigned as identified metabolites. Furthermore, metabolites with the highest similarity score were retained, whereas redundant metabolites were removed. In addition, only metabolites with a similarity score higher than 250 were kept for further quantification. To quantify the alteration of metabolites, missing data from when a metabolite was not detected by GC-MS were filled by the minimum number (0.0000000697) in this dataset.

### Quantification of amino acids

Free amino acids (AAs) were analysed according to the method of Wang *et al.* (2014) with some modifications. Filling grains were ground in liquid nitrogen and 50 mg was used for each sample. AAs were profiled on a Hitachi-L8900 Amino Acid analyser at the Instrumental Analysis Center of Shanghai Jiaotong University. Three biological replicates were performed. The dynamic profiles of free AAs were classified using the bioinformatics software GeneCluster 2.0 (Reich *et al.*, 2004). The concentration of AAs in grains was calculated relative to the sample fresh weight as µmol kg^–1^.

### Real-time quantitative RT-PCR

About 60 mg of filling grains (from at least three grains) were ground in liquid nitrogen. Total RNA was extracted with a RNA isolation kit (TianGene, Beijing, China) according to the manufacturer’s instructions. Real-time quantitative PCR was performed using a FastQuant RT kit and SuperReal PreMix Plus kit (TianGene, Beijing, China) followed the corresponding instructions. Three biological replicates were performed

### Measurement of ATP

ATP was isolated by trichloroacetic acid and detected using an ENLITEN ATP assay kit (Promega) as previously described (Xu *et al.*, 2008). Briefly, 2–3 immature grains were ground to a fine powder in liquid nitrogen. Then, 50 mg of the powder was transferred into a 1.5-ml Eppendorf tube and 200 μl of 5% trichloroacetic acid (w/v) was added for ATP extraction. Each sample was measured with biological replicates.

### Bioinformatic analysis

For proteomic data, HRPs were assigned to different biological processes with reference to the UniProt-GOA database (www.ebi.ac.uk/GOA/). Enrichment analysis was performed as described by Lu *et al.* (2017). For the metabolomic data, metabolites were assigned to different pathways following the KEGG pathway reference (http://www.kegg.jp/kegg/pathway.html). Enrichment analysis was performed using the software Metaboanalyst 2.0 (www.metaboanalyst.ca).

## Results

### General effects of heat on grain filling in the field

**Fig. 1.**
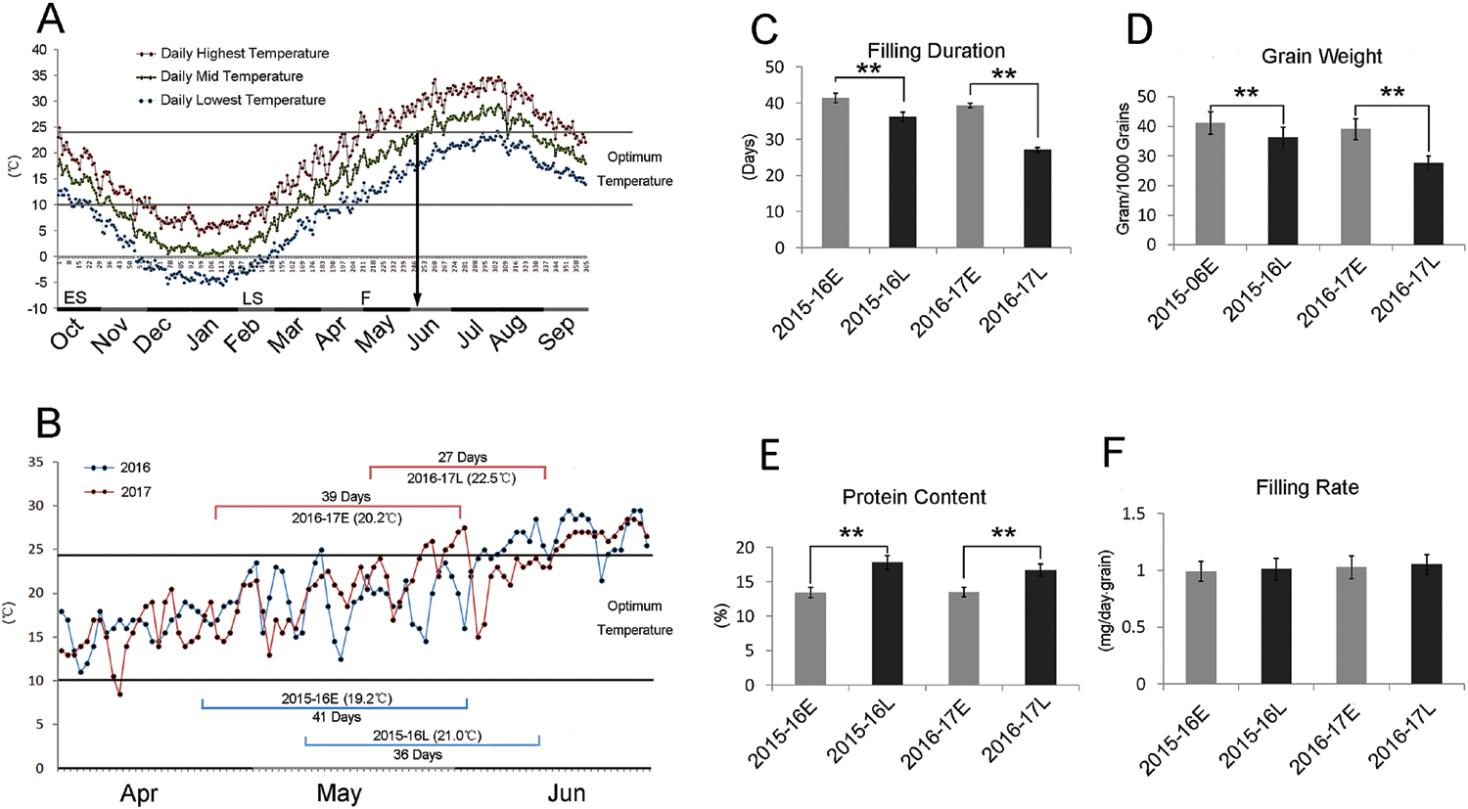
Effects of heat stress on filling wheat grains in the field. (A) Average air temperatures during the growing season for the years 2013–2017 at the experimental site in Yangling, China. The data show the mean daily highest, lowest, and mid-temperatures (i.e. mean of the highest and lowest). The two horizontal lines indicate the optimum temperature range for the grain-filling process. The arrow indicates the date at which the mid-temperature exceeded the optimum temperature for wheat grain filling, and most local varieties reach maturity by this time. The axis at 0 °C shows time in days. ES, early sowing time. LS, late sowing time; F, flowering time. (B) Temperature variation during the grain-filling stage in the two experimental growing seasons for early (E) and late (L) sowing. The average mid-temperature for each filling stage is indicated together with the average duration of the stage. (C–F) Filling duration, grain weight, protein content, and grain filling rate for 38 varieties. Data are means (±SD) based on six replicates per variety. Significant differences were determined using two-tailed, paired sample comparison *t*-tests: **P<0.01. (C) Filling duration is reduced with late sowing. (D) Grain weight is decreased with late sowing. (E) Grain protein content is increased with late sowing. (F) Filling rate is unchanged with late sowing. Filling rate was calculated as (grain weight)/(filling duration).

Bread wheat is usually sown in autumn in China (around October), such that emerging seedlings experience a cold winter; plants then reach the flowering stage in mid-spring of the following year and start to deposit grain reserves until maturity (termed the grain-filling stage). For most wheat varieties, optimum temperatures for filling are between 10– 24 °C (Zhao *et al.*, 2007; Farooq *et al.*, 2011). Temperatures rise in the summer (Fig. 1A) and this can cause heat stress during grain filling, and plants with a later filling stage are likely to suffer more intensive heat stress. Late-sown wheat has a later filling stage compared with early-sown wheat, and hence late sowing has been adopted as a method to induce heat stress in the field (Chauhan *et al.*, 2009; Tiwari *et al.*, 2012).

To investigate the effects of heat stress on filling grains, 38 wheat varieties/accessions previously identified as having different levels of thermotolerance were intensively studied using this approach of different sowing times across two growing seasons (2015–16 and 2016–17, Supplementary Table S1). In these two seasons, late sowing resulted in a filling stage postponed by 2– 3 weeks and resulted in an increase of ~2 °C in average temperature during the filling stage (Fig. 1B). In addition, late sowing significantly reduced the duration of grain filling by about 1–2 weeks, consistent with previous studies showing that heat reduces the duration (Farooq *et al.*, 2011). Notably, the average temperature during the filling stage in the 2016–17 growth season was 1.0–1.5 °C higher than that in 2015–16, suggesting that more intensive heat stress occurred. Consistent with this, the filling duration for both early- and late-sown wheat in 2016–17 was shorter than that in 2015–16 (Fig. 1B, C).

All the varieties sown late showed reduced filling duration times compared with those sown early for both the growing seasons, with the reduction varying from 2–9 d among the varieties (Fig. 1C, Supplementary Table S1). In the 2015–16 season, 37 out of the 38 varieties showed reduced grain weight in the late sowing compared with the early sowing, with an average reduction of 11.9% and ranging from 3–25% (Fig. 1D, Supplementary Table S1). In 2016–17, all the varieties sown late showed a significant decrease in grain weight compared with early sowing, with an average reduction of 29.5% and ranging from 12–40%, consistent with the more severe heat stress that occurred in that season (Fig. 1B, D). In addition, all of the varieties in the late sowing showed an increased protein content compared with the early sowing, with an average increase of 32.8% in the 2015–16 season and 23.6% in the 2016–17 season (Fig. 1E). However, the general filling rate, calculated as grain weight divided by the number of filling days, was not affected by heat stress in either of the seasons (Fig. 1F, Supplementary Table S1), although individual varieties showed some variation.

Collectively, the results from the field study showed that the general effects of heat stress were that grain weight and filling duration time decreased, the protein content increased, and the filling rate was not affected.

### Effects of heat on grain filling in greenhouse experiments

**Fig. 2.**
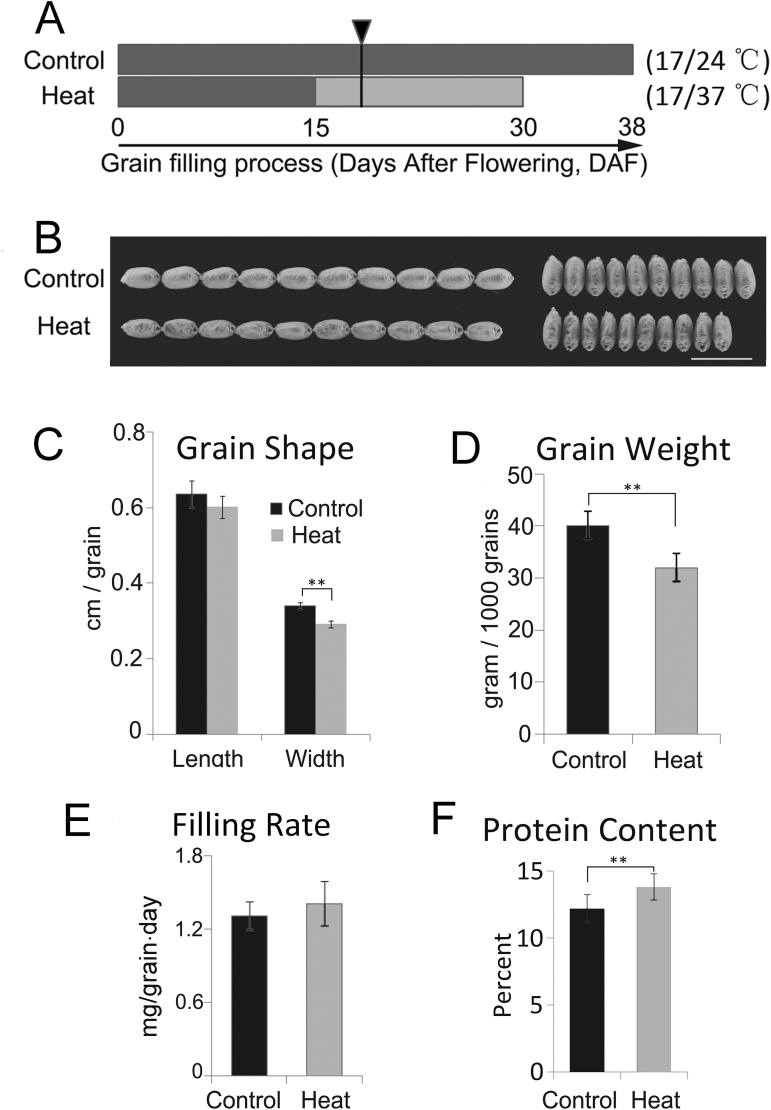
Grain development in wheat variety Chinese Spring subjected to heat stress in growth chambers. (A) Schematic diagram of the growth conditions, heat treatment, and sampling scheme. The heat stress regime was applied during grain filling starting at 15 d after flowering (DAF). Samples for metabolomic and proteomic analysis were collected at 18 DAF (black triangle). Note that grains reach maturity at 30 DAF and 38 DAF under the heat-stress and control conditions, respectively. A minimum of 20 plants were evaluated for each treatment. (B) Mature grains from heat-stressed plants are small and have a wrinkled coat. Scale bar is 1 cm. (C) Grain width is decreased by heat stress. (D) Grain weight is decreased by heat-stress. (E) Filling rate is remains relatively stable in heat-stressed filling grains. Filling rate was calculated as (grain weight)/(filling duration). (F) Protein content is increased in heat-stressed grains. In (C–F) a total of 200–300 grains from 20 spikes for both heat-stressed and control plants were measured. Significant differences compared with control conditions were determined using *t*-tests: **P<0.01.

To confirm that the effects of late sowing were the result of heat stress, we imposed a controlled daylight heat-stress regime on plants grown in the greenhouse (Fig. 2A). For these experiments, we selected the variety Chinese Spring, which was in the mid-range in terms of heat sensitivity among the varieties examined in the field study (Supplementary Table S1). Under the regime used, grains subjected to heat stress reached maturity at 30 d after flowering (DAF), while grains under control conditions reached maturity at 38 DAF (Fig. 2A). Mature grains from the heat treatment showed a significant reduction in width (Fig. 2B, C), and grain weight also decreased significantly (Fig. 2D), showing a 20% decrease corresponding to a reduction of 21% in the filling duration. In contrast, the average daily filling rate was relatively stable (Fig. 2E). These results confirmed that the loss in grain weight observed in the Chinese Spring variety in the field study mainly resulted from a reduction in the filling duration and that the protein content in mature grains actually increased after heat stress (by ~10%) (Fig. 2F).

### Proteomic analysis demonstrates significant decreases in protein synthesis components during heat stress

**Fig. 3.**
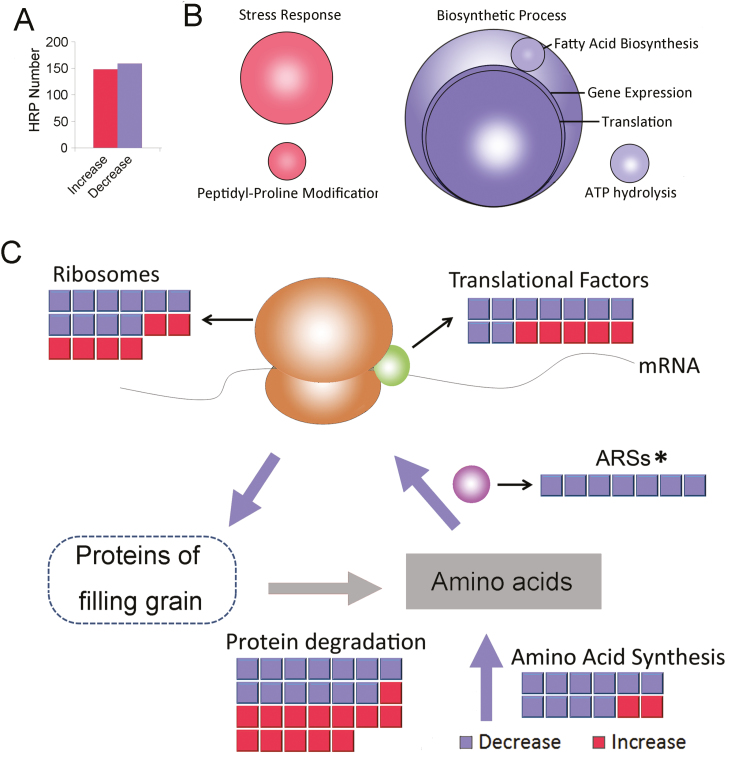
Proteomic analysis of the effects of heat stress on filling grains. (A) The numbers of heat-responsive proteins (HRPs) that were increased and decreased in response to heat stress. A full list of HRPs and category information are given in Supplementary Table S4. (B) Enrichment analysis of HRPs based on the KEGG process classification (Supplementary Table S5. Red circles represent significantly enriched processes in the increased HRPs and blue circles represent significantly enriched processes in the decreased HRPs (Fisher’s exact test, *P*<0.01). The size of each circle is proportional to the number of HRPs assigned to each process. (C) Schematic diagram showing changes in HRPs related to protein synthesis and degradation: red, increased; blue, decreased. The large arrows represent fluxes of amino acids, with blue indicating a decrease and grey indicating that the change in flux is uncertain. The circle with the dashed line represents the products of protein synthesis The asterisk for ARSs (aminoacyl tRNA synthetase) indicates a significant reduction as determined by a Chi-square test, *P*<0.05.

To investigate the molecular basis of the observed heat-induced alterations, filling grains at 15 DAF were subjected to 3 d of heat stress and then sampled for tandem mass tag (TMT)-labelled, quantitative proteomic analysis (Fig. 2A). A total of 5172 wheat proteins were identified (Supplementary Table S2). Of these, 1161 highly reliable proteins could be quantified (Supplementary Table S3), and 309 proteins were assigned as heat-responsive proteins (HRPs; fold-change >1.2 or <0.833, *P*<0.01, Fig. 3A, Supplementary Table S4). As expected, many heat-shock proteins (HSP70s, HSP40s, and HSP20s) and redox regulatory proteins, which were categorized as ‘stress response’ components, were significantly enriched in the increased HRPs (Fig. 3B, Supplementary Table S5). Decreased HRPs were significantly enriched in the category ‘biosynthetic processes’, including fatty acid biosynthesis and in particular protein synthesis (termed ‘gene expression’ and ‘translation’ in Fig. 3B, Supplementary Table S5), suggesting a global decrease in protein synthesis components. In addition, the category ‘ATP hydrolysis process’ was also enriched in decreased HRPs.

The HRPs involved in protein synthesis were further analysed (Fig. 3C), revealing that most ribosomes and translation factors (including 11 initiation factors) were significantly decreased in abundance in response to heat stress, consistent with previous reports of decreases in translational factors during heat stress (Spriggs *et al.*, 2010; Yamakawa and Hakata, 2010; de Nadal *et al.*, 2011). Notably, seven aminoacyl tRNA synthetases (ARSs), which catalyse the formation of the precursors (tRNA aminoacylation) and control the amino acid (AA) flux into protein synthesis (Ibba and Söll, 2000), showed a significant decrease (Chi-square test, *P*<0.05) during heat stress (Fig. 3C). This suggested that the process of AA flux into protein synthesis may have been impeded under heat stress, which provides further evidence for inhibition of protein synthesis in addition to the well-established decrease in translational factors.

### Increases in stress-response and storage proteins accompanied by a decrease in metabolic proteins during heat stress

**Fig. 4.**
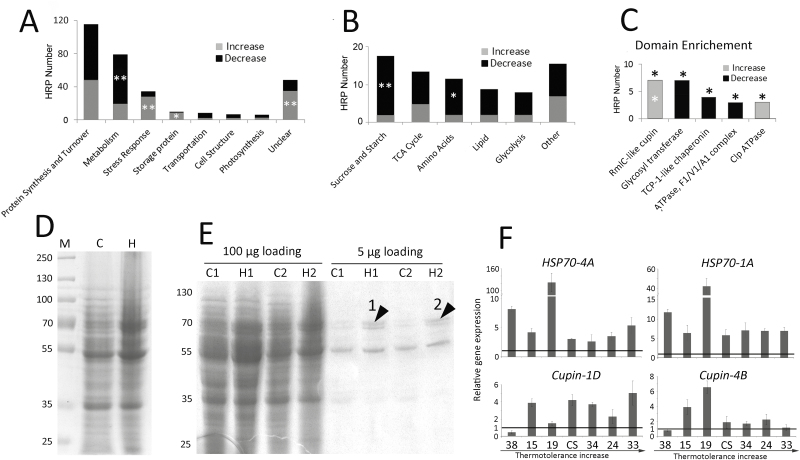
Category analysis of heat-responsive proteins (HRPs) and increased storage proteins in mature grains of the wheat variety Chinese Spring subjected to heat stress under controlled conditions. (A) Distribution of HRPs among different categories. HRPs in the category ‘Metabolism’ are decreased, whereas those in the categories ‘Stress response’ and ‘Storage protein’ are increased as a result of heat stress (Chi-square test: *P<0.05, **P<0.01). The full list of category classifications of HRPs is shown in Supplementary Table S4. (B) Distribution of HRPs in each sub-category of ‘Metabolism’. (C) Enriched protein domains in HRPs. Asterisks above the bars indicate that the domain was significantly enriched in HRPs (Fisher’s exact test, *P*<0.05) and an asterisk inside the bar indicates that proteins with the domain were significantly increased or decreased (Chi-square test, *P*<0.05). (D) Proteins with mass ~70 kD were induced in the mature heat-stressed grains. Protein samples were extracted from mature grains with and without heat stress. Equal quantities of protein were loaded onto each lane. M, marker; C, control conditions; H, heat-stressed conditions. Numbers on the left indicate the molecular weight of the proteins (kD). (E) A band around 70 kD is dramatically induced in mature heat-stressed grain. C1 and C2 refer to two independent samples from the control conditions, and H1 and H2 refer to two independent samples from the heat-stressed conditions. Bands 1 and 2 (marked with arrowheads) were selected for MS analysis to identify and quantify the storage proteins in these bands (see Methods). The MS results are shown in Supplementary Table S6. (F) The transcriptional response of *HSP70* and *Cupin* in wheat accessions with different levels of heat resistance (*x*-axis, see Supplementary Table S1). Gene expression is expressed relative to that of *Actin*. Gene names are assigned according to the gene and its chromosome location shown in Supplementary Table S4. Data are means (±SD) from three biological replicates. The horizontal lines represent a fold-change of 1, i.e. no change under heat stress.

To obtain a comprehensive overview of the alterations in proteins in heat-stressed grains, 309 HRPs were classified into nine categories based on the protein GO classification (Fig. 4A, Supplementary Table S4). HRPs involved in metabolism were found to be significantly decreased (Chi-square test, *P*<0.05); consistent with this, most HRPs in each sub-category of metabolism showed a decreased abundance, and the metabolic processes of ‘sucrose and starch’ and ‘amino acid’ showed a significant decrease (Chi-square test, *P*<0.05, Fig. 4B). In contrast, HRPs in the stress-response category, which includes chaperones, redox proteins, and other stress-responsive proteins (Supplementary Table S4), displayed a significant increase (Chi-square test, *P*<0.05). Of these, 11 out of 14 chaperones were increased in abundance, indicating that HSP synthesis may be exempt from the general heat inhibition of protein synthesis in order to protect and recover damaged proteins (Hendrick and Hartl, 1993; Wang *et al.*, 2004; Spriggs *et al.*, 2010).

Similarly, 10 HRPs that belong to storage proteins were also significantly enriched among the increased proteins (Chi-square test, *P*<0.01) (Supplementary Table S4). These included seven cupin proteins, which were defined based on their RmlC-like cupin domain and RmlC-like jelly roll fold, that shared high similarity with the wheat storage protein globulin 3 (>80% identity). Domain enrichment analysis showed that the RmlC-like cupin domain was significantly enriched in HRPs (Fig. 4C; Fisher’s exact test, *P*<0.05) and significantly skewed towards increased enrichment (Chi-square test, *P*<0.01). These results indicated that cupin protein synthesis was also exempt from the general inhibition of protein synthesis and it accumulated in heat-stressed grains, which was consistent with the increase in protein content in mature heat-stressed grains (Fig. 1E, 2F). Thus, cupin protein accumulation probably contributed greatly to the relative increase in the protein content of heat-stressed grains.

To confirm this hypothesis, total proteins from mature grains were separated by SDS–PAGE, and several bands of around 70 kD were found to be increased in the heat-stress treatment (Fig. 4D). These band changes were consistently observed in the different heat-stress replicates. After the dilution of the protein samples, a band of around 70 kD (Fig. 4E, black arrows) showed a consistent significant increase in mature heat-stressed grains. MS analysis of these two bands from different replicates identified cupin domain-containing proteins, including globulin 3, as the predominant component (>85%, Supplementary Table S6), strongly supporting the increase in cupin proteins during heat stress as substantially contributing to the increase in the storage protein content in mature grains. Given that the 70-kD band showed high abundance and was one of the three major bands in the heat-stressed protein samples (Fig. 4E), the cupin proteins may be the main heat-induced storage proteins and therefore responsible for the overall increased protein content.

To check whether cupin accumulation was a common heat response, seven wheat accessions with different thermotolerance were selected for evaluation. We found that two *HSP70*s were significantly induced at varying levels in all the accessions under heat stress, and the *Cupin*s were induced in most of the accessions (Fig. 4F), indicating that accumulation of cupins under heat stress was a widely distributed heat response in wheat grains.

### AAs accumulate in filling grains subjected to heat stress

**Fig. 5.**
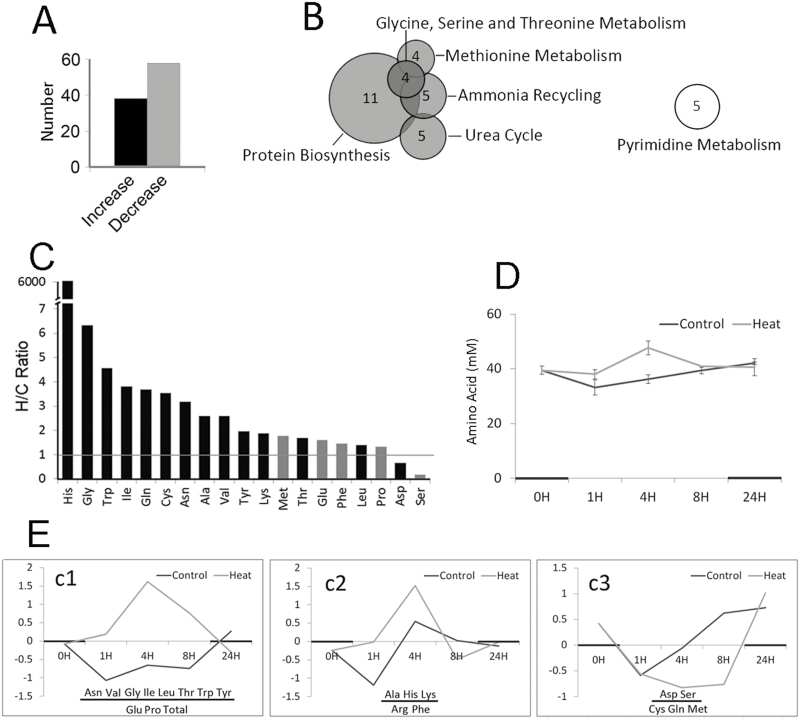
Changes in metabolites in heat-stressed filling grains of the wheat variety Chinese Spring subjected to heat stress under controlled conditions. (A) Numbers of increased and decreased metabolites in heat-stressed samples (see Supplementary Table S7 for detailed information). (B) Summary of enrichment analysis from the metabolomic data (see Supplementary Table S8 for detailed information). The grey circles represent significantly enriched processes in the increased metabolites and the white circle represents significantly enriched processes in the decreased metabolites (*P*<0.05, Fisher’s exact test). The number of identified metabolites in each process is indicated. (C) Fold-changes in amino acid (AA) contents in heat-stressed (H) and control (C) grain samples. Bars that are black indicate significant changes in heat-stressed grains (1.5-fold and *t*-test *P*<0.05; from the data in Supplementary Table S7). (D) Time-course of changes in total free AAs. Samples for quantification were taken 15 d after flowering (DAF) when the first heat stress cycle was applied (see Fig. 2A). The shading on the *x*-axis indicates the dark period in the growth chamber. Data are means (±SD) from three biological replicates. (E) Time-courses of changes for different classes of AAs. GeneCluster 2.0 was applied to classify the AA profiles. The AAs that are underlined showed the same changes as observed in the metabolomic analysis. The values on the *y*-axis are derived from GeneCluster analysis to represent the relative changes in AA content compared to samples with control temperature for each time-point.

To understand the metabolic alterations that occurred in grain filling during heat stress, the same samples used in the proteomic analysis were examined using GC-MS unbiased metabolomic analysis. This analysis assigned 98 out of 258 identified metabolites as significantly changed by the heat treatment (>1.5-fold, *t*-test *P*<0.05; Supplementary Table S7), with 60 decreased and 38 increased (Fig. 5A), which was consistent with the observed decrease in metabolic proteins. Metabolites involved in protein biosynthesis and AA metabolism were significantly enriched among the increased metabolites, whereas those involved in pyrimidine metabolism were enriched among the decreased metabolites (Fig. 5B, Supplementary Table S8).

It was notable that metabolites that were enriched in protein synthesis and AA metabolism included all types of proteinogenic AAs. We found that 13 out of 19 identified free proteinogenic AAs were significantly increased during heat stress (Fig. 5C; 1.5-fold, *t*-test, *P*<0.05). Given that AAs are highly dynamic metabolites (Mangelsen *et al.*, 2011), we further investigated free AA pools over a time course of 0–24 h during the first day of heat treatment (15 DAF). The total free AA content was significantly increased after 1 h and 4 h of heat stress (Fig. 5D, Supplementary Table S9). The time-course profiles of each AA could be divided into three classes using the GeneCluster 2.0 software (Fig. 5E). Classes c1 and c2 (15 AAs) comprised AAs that increased during heat stress, 11 of which also showed a significant increase in the metabolomic analysis. Class c3 (five AAs) included AAs that were found to be decreased or remained at stable levels during heat stress, further confirming the results observed for Ser and Asp in the metabolomic analysis (Fig. 5C). The data supported the conclusion that AA accumulation is a typical heat response and were consistent with the significant decrease in the abundance of ARSs and proteins in the protein synthesis component (Fig. 3C). However, these results contradicted the significant decrease of HRPs involved in AA biosynthesis and the global decrease in metabolism under heat stress (Fig. 4A, B). Given that protein degradation showed no detectable shift towards an increase or decrease during heat stress (Fig. 3C), our proteomic results suggested that the general increase in free AA content may have mainly resulted from the inhibition of protein synthesis.

### Metabolomic analysis demonstrates significant decreases in the metabolites of central carbohydrate metabolism

**Fig. 6.**
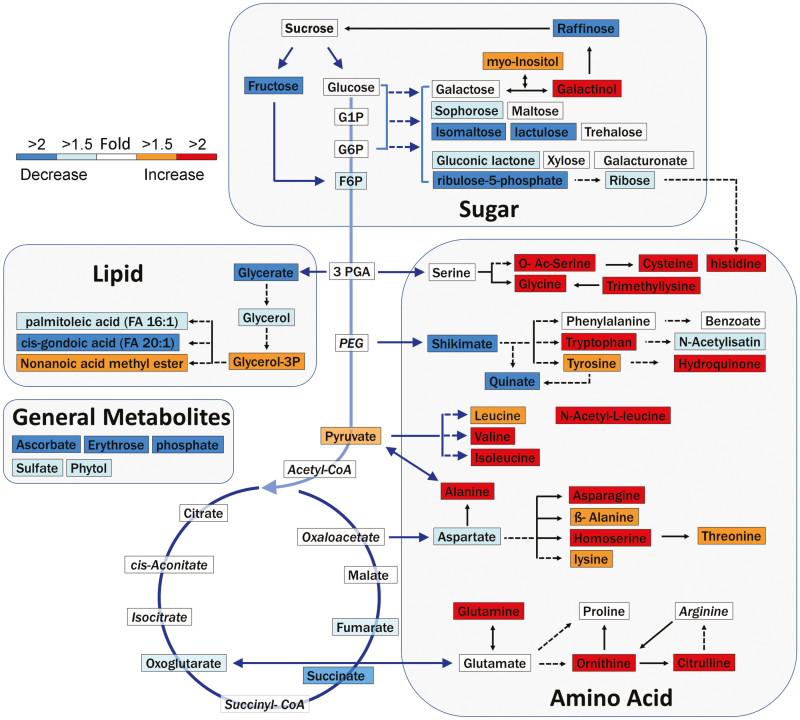
Changes in metabolites in response to heat stress. The identified metabolites were mapped to the carbohydrate metabolic pathways in the KEGG reference pathways. Red and blue boxes represent significantly increased and decreased metabolites, respectively (*t*-test, *P*<0.05). White boxes represent metabolites with no significant changes. Italics indicate metabolites that were not detected in this experiment. Arrows with solid lines indicate direct precursors and products, while arrows with dashed lines refer to indirect precursors and products.

To investigate global alterations in metabolism of carbohydrates, which are known to be the major component of assimilation flux in filling grains, we mapped the identified metabolites to sugar conversion, glycolysis, TCA cycle, and lipid metabolism (Fig. 6). We found that 16 out of 21 mapped metabolites in these carbohydrate metabolism pathways were decreased in response to heat stress. Similarly, several general metabolites, including free phosphate, sulphate, ascorbate, erythrose, and phytol, were also identified as metabolites changed by heat, and all were decreased. These metabolomic results indicated that less assimilation was allocated to these metabolic pathways during heat stress. However, G1P and G6P, the carbohydrate precursors for starch synthesis, remained stable during heat stress (Fig. 6, Supplementary Table S7), consistent with previous reports that sucrose and G1P levels are relatively stable in heat-stressed filling grains of wheat (Zahedi *et al.*, 2003).

### Energy-consuming HRPs demonstrate that energy is transferred from metabolism and protein synthesis to protein protection during heat stress

Energy is a critical factor that drives most irreversible biochemical reactions. Among the 309 HRPs that we identified, 47 were characterized as energy metabolism-related proteins based on the presence of ATP (or GTP) binding, kinase, and ATPase domains, including three ATP synthesis-related proteins (F-type ATPase) and 44 energy-consuming proteins (Table 1). All three ATP synthesis-related proteins were decreased, which was consistent with ATP metabolism enrichment in the decreased HRPs (Fig. 3B) and the decreased TCA-related metabolite content (Fig. 6). These data indicated a decrease in energy synthesis during heat stress, which is consistent with previous observations that energy synthesis is decreased in heat-stressed filling grains of wheat and rice (Majoul *et al.*, 2004; Neilson *et al.*, 2010; Yamakawa and Hakata, 2010; Glaubitz *et al.*, 2015).

**Table 1.** Details of energy-related heat-responsive proteins

No.	**Uniprot ID**	**KEGG gene**	**He**at**/Co**ntrol **ratio**^**†**^	**Domain**	**Biological process**
Metabolism					
1	A0A1D6RCT7	Fructokinase	0.76	Kinase	Sucrose metabolism
2	W5GA68	Diphosphate-dependent phosphofructokinase	0.72	Kinase	Glycolysis/Gluconeogenesis
3	A0A1D5RX09	Pyruvate, orthophosphate dikinase	0.76	Kinase	Glycolysis/Gluconeogenesis
4	A0A1D6D1D7	Pyruvate, orthophosphate dikinase	0.80	Kinase	Glycolysis/Gluconeogenesis
5	A0A1D5T5P0	Pyruvate, orthophosphate dikinase	0.74	Kinase	Glycolysis/Gluconeogenesis
6	W4ZUF2*	ATPeF1G	0.79	ATPase	TCA cycle
7	A0A1D6ACZ4*	ATPase, F0F1 subunit alpha	0.65	ATPase	TCA cycle
8	A0A1D6RG61*	ATPase, F0F1 subunit alpha	0.59	ATPase	TCA cycle
9	A0A1D5SUB6	Asparagine synthase	0.73	ATP binding	Amino acid synthesis
10	W5F6U4	Long-chain acyl-CoA synthetase	0.51	Ligase	Lipid metabolism
11	A0A1D5TLL5	Acetyl-CoA carboxylase	0.63	ATP binding	Lipid metabolism
12	A0A1D6BFQ9	L-arabinokinase	0.77	Kinase	Metabolism
13	A0A1D5UV37	Carbamoyl-phosphate synthetase	0.58	ATP binding	Metabolism
14	A0A1D5UVH8	D-alanine–D-alanine ligase	0.65	Ligase	Metabolism
Protein synthesis					
15	A0A1D5Y4B3	Alanyl-tRNA synthetase	0.80	Ligase	Translation
16	W5FYH5	Aspartyl-tRNA synthetase	0.75	Ligase	Translation
17	A0A1D6BHS4	Isoleucyl-tRNA synthetase	0.65	Ligase	Translation
18	A0A1D6RRI1	Qlutaminyl-tRNA synthetase	0.66	Ligase	Translation
19	A0A1D5WWM5	Threonyl-tRNA synthetase	0.66	Ligase	Translation
20	A0A1D6RUT6	Valyl-tRNA synthetase	0.79	Ligase	Translation
21	A0A1D5VRW7	Valyl/Leucyl/Isoleucyl-tRNA synthetase	0.75	Ligase	Translation
22	W5GF95	Translation initiation factor 4A	0.61	ATP binding	Translation
23	A0A1D5ZWW5	Elongation factor Tu	0.76	GTP binding	Translation
Protein degradation					
24	A0A1D6BI38	26S proteasome regulatory subunit T4	0.66	ATPase	Protein degradation
25	A0A1D5SA32^#^	Clp ATPase	1.60	ATPase	Protein degradation
26	A0A1D5Y3B7^#^	Clp ATPase	1.42	ATPase	Protein degradation
27	A0A1D6BE72^#^	Clp ATPase	1.54	ATPase	Protein degradation
Stress response					
28	A0A1D6BHW3	Heat shock protein 90	0.62	ATPase	Heat stress
29	A0A1D5ZCZ5	Heat shock protein 90	0.61	ATPase	Heat stress
30	A0A1D5RYQ8	Heat shock 70k	1.73	ATP binding	Heat stress
31	A0A1D5WAT5	Heat shock 70k	3.34	ATP binding	Heat stress
32	W5DYF8	Heat shock 70k	3.74	ATP binding	Heat stress
33	A0A1D5S1X4	Heat shock 70k	1.44	ATP binding	Heat stress
34	A0A1D5U1X8	Heat shock 70k	0.60	ATP binding	Heat stress
25	A0A1D5SA32^#^	Clp ATPase	1.60	ATPase	Heat stress
26	A0A1D5Y3B7^#^	Clp ATPase	1.42	ATPase	Heat stress
27	A0A1D6BE72^#^	Clp ATPase	1.54	ATPase	Heat stress
Other					
35	A0A1D6S7C8	Phosphoribulokinase	0.69	Kinase	Photosynthesis
36	P12782	Phosphoglycerate kinase	0.73	Kinase	Photosynthesis
37	W5DSM4	RCA	1.22	ATPase	Photosynthesis
38	A0A1D5VS96	ATPeV1B	0.78	ATPase	Transportation
39	Q2L9B8	ATPeV1E	0.83	ATPase	Transportation
40	A0A1D6RWC8	P-type ATPase	0.75	ATPase	Transportation
41	P83970	P-type ATPase	0.74	ATPase	Transportation
42	A0A1D6BP32	chlI; magnesium chelatase subunit I	0.76	ATPase	Protein export
43	A0A096UQM6	ABC transporter-like	1.23	ATPase	Protein export
44	A0A1D5Y2P6	Signal recognition particle receptor	0.76	ATPase	Signalling
45	A0A1D5SGI0	Serine/threonine-protein kinase	0.66	Kinase	Protein modification
46	W4ZT36	DNA replication licensing factor MCM2	0.81	ATP binding	Cell structure
47	W4ZZM2	Structural maintenance of chromosomes protein	0.66	ATP binding	Cell structure

^†^ The Heat/Control ratio represents the change in protein abundance under heat stress compared with control conditions. * Proteins related to ATP synthesis. ^#^ Proteins classified into two categories.

**Fig. 7.**
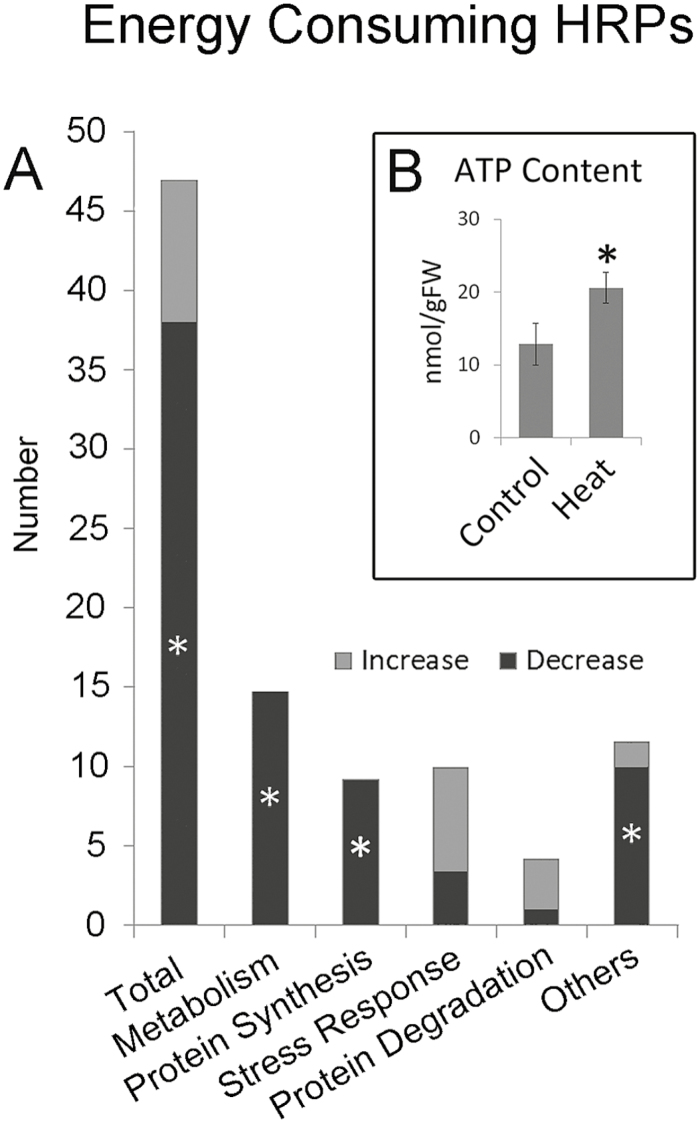
Calculation of estimated energy levels and costs during grain filling under control and heat-stress conditions in wheat variety Chinese Spring. (A) Energy-consuming heat-responsive proteins (HRPs). Asterisks indicate that the HRPs in each category show a significant increase or decrease (Chi-square test, *P*<0.05). (B) ATP levels in grains from control and heat-stressed conditions; * *P*<0.05 (*t*-test). Data are means (±SD) determined from three independent samples.

For the energy-consuming HRPs, 35 out of 44 were decreased under heat stress (Fig. 7A; Chi-square test, *P*<0.05, Table 1), indicating the lower energy-consuming status of filling grains. As protein synthesis is a major energy-consuming process in the cell (Li *et al.*, 2014), we evaluated the ATP-consuming HRPs involved in protein synthesis. We found that all nine energy-consuming HRPs in this category were decreased, including all seven ARSs and two translational initiation/elongation factors, suggesting that there was decreased energy consumption for protein synthesis during heat stress. Similarly, all 13 energy-consuming HRPs in metabolic pathways were significantly decreased, indicating that energy may have been saved by reducing the activity of these pathways. It was notable that energy-consuming HRPs both in metabolism and in protein synthesis showed a significant decrease (Fig. 7A; Chi-square test, *P*<0.05). In contrast, seven out of ten energy-consuming proteins that were identified as increased HRPs were enriched in the stress-response category (Table 1), suggesting that more energy may have been channelled into stress resistance under heat stress.

These alterations in energy-consuming HRPs clearly demonstrated that more energy was transferred from metabolism and protein synthesis to stress resistance during heat stress. However, surprisingly, the ATP concentration was increased under heat conditions (Fig. 7B), implying that the decreases in metabolic and protein-synthesis processes utilized less energy, resulting in an ATP increase in heat-stressed filling grains.

Overall, our proteomic and metabolic data provided parallel and consistent evidence that the proteins involved in metabolic and protein-synthesis processes were generally decreased, and then adaptive metabolism reallocated assimilation into the deposition of reserves in order to maintain a stable grain-filling rate under heat stress. Correspondingly, energy consumption was transferred to protein protection from metabolism and protein synthesis, suggesting a global heat-adaptive metabolism that conferred lower assimilation/energy consumption and higher thermostability in order to adapt to heat stress as well as maintaining the key filling process in the stressed grains.

## Discussion

The grain-filling process in wheat is regarded as a critical stage for maintaining yield; however, this process is threatened by rising summer temperatures, especially under global-warming scenarios. Our field study demonstrated that different wheat varieties showed different degrees of alterations in response to the same heat stress, consistent with previous studies (Stone and Nicolas, 1994; ur Rehman *et al.*, 2009; Bahar and Yildirim, 2010), indicating that current varieties harbour genetic diversity that can provide the resources for breeding better thermotolerance (Yeh *et al.*, 2012). On the other hand, the same varieties showed different alterations in response to different heat regimes, suggesting that heat imposes a complex and dynamic stress (Farooq *et al.*, 2011) and its effects depend on the extent of the stress, which makes it difficult to predict the outcome for any given variety. However, our results confirmed that reduced filling duration, decreased grain weight, and increased protein content were common alterations detected across diverse varieties under different heat regimes.

The daily filling rate remained relatively stable under all heat-stress conditions investigated, indicating that the daily deposition of reserves was not affected. Starch accumulation, which is usually the major component of deposition, was impeded during heat stress, and the proteomic data indicated that this may have resulted from decreases in three starch synthesis-related proteins (two starch synthases and a granule-bound starch synthetase) (Supplementary Table S4). In contrast, storage proteins, another major grain reserve, were significantly increased during heat stress, supporting the hypothesis that their accumulation is a critical mechanism for maintaining the filling rate (Farooq *et al.*, 2011). Consistent with previous reports, however, the precursors of starch synthesis, G1P and G6P, remained relatively stable under heat stress, in contrast to the overall decrease in carbohydrate metabolites, which may be another critical mechanism that maintains the rate of starch synthesis and may also contribute to a stable filling rate.

The increase in the protein content in mature heat-stressed grains further suggested that more energy and greater assimilation were allocated to the deposition of protein reserves, despite the fact that the data indicated an overall decrease in protein synthesis during stress. Cupins were identified as major heat-responsive storage proteins and these made a substantial contribution to the increased protein content in mature grains. Proteins containing a cupin domain were originally identified in wheat grains with an unusual thermostable character, and tend to accumulate in a number of heat-stressed organisms (Dunwell *et al.*, 2001). The thermostable character of cupins may facilitate their deposition during heat stress. In wheat grains, cupin-containing proteins usually accumulate at late developmental stages, and they have been proposed to have a stress-response function (Arena *et al.*, 2017); however, their exact biological function requires further study. Furthermore, how these proteins are preferentially accumulated when protein synthesis components are generally decreased during heat stress needs to be investigated, and this may provide valuable insights into improving the protein content of wheat.

Accumulating evidence has shown that processes required for energy production are significantly decreased during heat stress (Majoul *et al.*, 2004; Yamakawa and Hakata, 2010; Li *et al.*, 2011; Mangelsen *et al.*, 2011). However, we found that the ATP status was actually increased by heat-stress, questioning our understanding of energy metabolism in filling grains. In this study, the channelling of energy was evaluated by investigating energy-consuming HRPs, and the results were consistent with the general proteomic and metabolic data, indicating that the increased ATP status in heat-stressed grains may have resulted from the general decrease in energy-consuming HRPs, thus providing a different perspective into specific alterations in heat-stressed filling grains.

In summary, our study uncovered alterations in metabolism in heat-stressed filling wheat grains, which produced apparent advantages in terms of conserving energy and limiting assimilation, whilst instead transferring efforts towards the deposition of reserves (starch and storage proteins) and protein protection. These findings provide insights into the molecular basis for alterations caused by heat stress in filling grains. Furthermore, our identification of accumulation of cupin proteins, energy-channelling, and a higher energy supply status also contributes to our understanding of heat adaptation in filling grains. Our data provide a foundation for understanding how metabolism is altered in filling grains during heat stress, which will facilitate the design of strategies to improve wheat grain quality via genetic and breeding approaches.

## Supplementary data

Supplementary data are available at *JXB* online.

Table S1. Data from the field study of responses to heat stress in 38 varieties of wheat.

Table S2. Peptides acquired by TMT-labelled proteomics.

Table S3. Protein identification and quantification.

Table S4. Identification of heat-responsive proteins and functional classification.

Table S5. Enrichment analysis of heat-responsive proteins.

Table S6. Identification and quantification of increased storage proteins.

Table S7. Results of the metabolomic analysis.

Table S8. Enrichment analysis of metabolites changed in response to heat stress.

Table S9. Time-course of amino acid profile analysis in response to heat stress.

Supplementary Tables S1-S9

## Data deposition

Proteome MS data has been deposited to the ProteomeXchange Consortium via the PRIDE database under the title ‘Heat-adaptive proteome in filling wheat grain’. doi:10.6019/PXD010340 (Wang *et al.*, 2018).

## Abbreviations:

ARS: aminoacyl tRNA synthetase
DAF: days after flowering
TMT: tandem mass tag
HRP: heat-responsive protein
